# Cilia Provide a Platform for the Generation, Regulated Secretion, and Reception of Peptidergic Signals

**DOI:** 10.3390/cells13040303

**Published:** 2024-02-06

**Authors:** Raj Luxmi, Stephen M. King

**Affiliations:** Department of Molecular Biology and Biophysics, University of Connecticut Health Center, 263 Farmington Avenue, Farmington, CT 06030-3305, USA; luxmi@uchc.edu

**Keywords:** amidation, *Chlamydomonas*, cilia, ectosome, peptidergic signaling, secretion

## Abstract

Cilia are microtubule-based cellular projections that act as motile, sensory, and secretory organelles. These structures receive information from the environment and transmit downstream signals to the cell body. Cilia also release vesicular ectosomes that bud from the ciliary membrane and carry an array of bioactive enzymes and peptide products. Peptidergic signals represent an ancient mode of intercellular communication, and in metazoans are involved in the maintenance of cellular homeostasis and various other physiological processes and responses. Numerous peptide receptors, subtilisin-like proteases, the peptide-amidating enzyme, and bioactive amidated peptide products have been localized to these organelles. In this review, we detail how cilia serve as specialized signaling organelles and act as a platform for the regulated processing and secretion of peptidergic signals. We especially focus on the processing and trafficking pathways by which a peptide precursor from the green alga *Chlamydomonas reinhardtii* is converted into an amidated bioactive product—a chemotactic modulator—and released from cilia in ectosomes. Biochemical dissection of this complex ciliary secretory pathway provides a paradigm for understanding cilia-based peptidergic signaling in mammals and other eukaryotes.

## 1. Introduction

Cilia are membrane-bound cellular projections built around a microtubule scaffold. In addition to their well described role in the generation of cell motility and fluid flow, these organelles have emerged as a vital hub for a diverse array of signaling pathways. Cilia act as cellular antennae, receiving signals from the extracellular environment and transducing information to the cell body [1,2]. Studies in a broad phylogenetic array of organisms, including the green alga *Chlamydomonas reinhardtii*, the nematode *Caenorhabditis elegans*, and mammals, revealed that cilia secrete ectosomes through budding of the ciliary membrane [3,4,5,6]. These extracellular vesicles contain bioactive enzymes and peptide products that can mediate degradative processes, intercellular and inter-organismal communication, and other diverse aspects of organismal physiology [3,5,6,7,8,9]; potentially, these vesicles might even act in an autocrine manner.

Peptide hormones serve as critical messengers in cellular communication, regulating a vast array of physiological processes. These peptides are synthesized from large inactive precursors in the lumen of the secretory pathway and can be stored in secretory granules, which are released in response to appropriate stimuli into the surrounding environment [10,11]. Peptide secretion is tightly controlled and precisely timed. Many peptide receptors, such as those binding somatostatin, neuropeptide Y, and α-melanocyte-stimulating hormone, localize to cilia [12,13,14]. Cilia-based signaling has been implicated in critical physiological and pathological phenomena, ranging from embryonic development and tissue homeostasis to diseases such as polycystic kidney disease and various ciliopathies [8,15,16].

Many peptides require the conversion of a C-terminal glycine residue into an α-amide to be bioactive; this reaction was first studied in secretory granules [17]. It is one of the final steps in the synthesis of signaling peptides and is catalyzed only by the peptide-amidating enzyme, peptidylglycine α-amidating monooxygenase (PAM). In metazoans, it is well known that many secreted peptides, such as neuropeptide Y, vasopressin, and oxytocin, are α-amidated at the C-terminus. This specific post-translational modification makes the peptide less sensitive to physiological pH changes and less vulnerable to proteases, as they are poorly recognized by carboxypeptidases and can enhance the affinity for binding to their cognate receptor by several orders of magnitude (reviewed in [18,19]). In addition to regulating mammalian physiology, α-amidated peptides are used in defense responses, being found in various toxins/venoms produced by a wide variety of metazoans such as wasps, snakes, cone snails and frogs [20,21,22]; they also regulate the behavior of numerous organisms such as chlorophyte algae [5], placozoans [23], *Drosophila*, sea urchins, and marine annelids [24,25,26]. Importantly, both PAM and amidated products localize to cilia and are secreted into the environment on ciliary ectosomes. For example, in *C. reinhardtii*, an amidated product (GATI-*amide*) that is synthesized from a large proGATI precursor and secreted from cilia acts as a chemotactic modulator, ultimately controlling the ciliary motility of gametic cells [5,27,28].

Here, we provide a comprehensive exploration of the cilia-based peptidergic signaling paradigm, i.e., how cilia can act as a specialized signaling device for both the production and regulated secretion of bioactive peptides and the perception and transduction of peptidergic signals. We discuss the processing pathway for an amidated peptide precursor, proGATI, in cilia, and the regulated secretion of its bioactive products in ciliary ectosomes. As components of this complex processing scheme are highly conserved throughout the eukaryotes, detailed understanding of this pathway in *C. reinhardtii* sheds light on how a ciliary peptide precursor may be processed and subsequently signals through ciliary ectosomes in metazoans.

## 2. General Organization of Cilia

Cilia are membrane-bound cellular projections with a core made of a microtubule-based cytoskeleton known as the axoneme. Although in some cells, ciliary structures have been highly modified to perform unique cellular functions, their structural framework has in general been very highly conserved [29,30]. Axonemal doublet microtubules derive from the triplet microtubules of the basal body, which is located in the cytoplasm and provides the structural template for ciliary formation.

Classically, cilia are divided into two classes—motile and primary (signaling)—although it is now clear that motile cilia also act as sensors and signaling platforms. Usually, motile cilia have a 9+2 microtubule arrangement, with nine doublet microtubules arranged in a ring and surrounding a central pair of singlet microtubules [29], although variations on this theme do occur in some organisms (e.g., the 9+4 organization of cilia on the notochordal plate of rabbit embryos [31]; the variable (9v) doublet microtubule arrangement in the parasite *Leishmania mexicana* [32]; and the 3+0 axonemal structure of the parasitic protozoan *Diplauxis* [33,34]). The central pair of microtubules are adorned with a complex array of protein protrusions and connect to the outer doublet microtubules via the radial spokes, which are thought to transmit signals controlling the activity of the inner and outer rows of dynein arms that power ciliary beating [35].

In mammals, motile cilia are present in various cell types, including sperm and the ciliated epithelial cells that line the airways, the ependyma in the brain ventricles, and the oviduct [36]. There are also specialized motile cilia lacking the central pair complex located at the embryonic node; their vortical motion is needed to establish the left–right body axis during development [37,38]. In addition, nearly all cells at some point in their life cycle bear non-motile primary cilia that function in a broad array of signaling pathways, e.g., hedgehog signaling [16]. Although originally considered to have a 9+0 microtubule organization, tomographic reconstructions of mammalian primary cilia have revealed that the situation is more complex and irregular. The 9+0 arrangement only occurs near the ciliary base and then changes to a crosslinked bundle with the microtubule number decreasing towards the ciliary tip [39].

**Figure 1:**
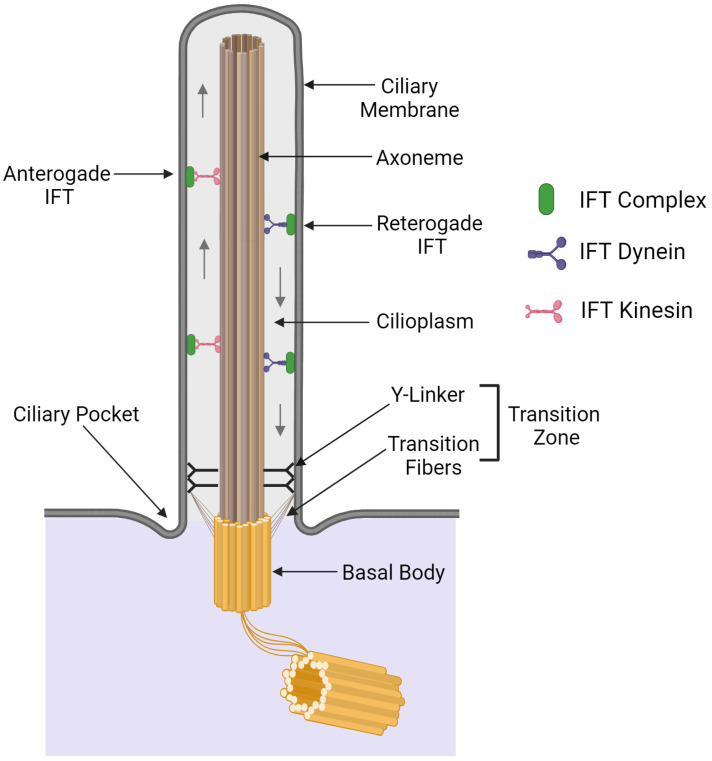
Generic schematic of 9+0 ciliary organization. The cilium comprises a microtubular axoneme that derives from the triplet microtubules of the basal body. The axoneme is surrounded by the ciliary membrane, which is contiguous with, but has a different composition from, the plasma membrane. The transition zone at the ciliary base contains Y-linkers and transition fibers; this structure acts as a gate to control what components can enter the cilium. Cilia assemble at their distal tip, and many components are trafficked there by the IFT system. The movement of IFT particles towards the ciliary tip (anterograde transport) is driven by a heterotrimeric kinesin, while a distinct dynein complex (the IFT dynein) returns them to the ciliary base (retrograde transport). The illustration was created with BioRender.com.

Ciliary assembly and maintenance are mediated by the bi-directional intraflagellar transport (IFT) of protein cargoes along the length of the cilium (Figure 1) [40]. This complex multi-component system involves the movement of protein trains consisting of both IFT particle scaffolds and cargoes along the doublet microtubules: anterograde transport is mediated by a heterotrimeric kinesin II motor, while a specialized dynein returns remodeled trains to the ciliary base [41]. Defects in IFT components can lead to the partial or even complete failure of ciliary assembly, with concomitant defects in cilia-based signaling [42,43].

The transport of proteins into and out of the cilium is tightly regulated by a diffusion-based barrier known as the transition zone (TZ); this structure is functionally analogous to nuclear pores which control passage into and out of the nucleus. The TZ is located at the base of the cilium and characterized by the presence of Y-shaped linkers (Y-linkers) that connect outer microtubule doublets of the axoneme to the ciliary membrane (Figure 1) [44]. Transition fibers are anchored to the basal body and connect to the ciliary membrane. At the TZ, microtubule triplets of the basal body are extended as the doublet microtubules of the axoneme [45]. Collectively, transition fibers and Y-linkers act as a gate to control the entry and exit of ciliary proteins and the passage of specific lipids to the ciliary membrane [46]. The TZ houses several protein complexes, including the nephronophthisis (NPHP) and the Meckel syndrome (MKS) complexes, which play crucial roles in ciliary trafficking and maintaining signaling functions. Mutations in these proteins can result in ciliopathies—syndromes with a broad array of phenotypes affecting multiple organ systems [47,48].

The ciliary axoneme and soluble matrix components are enclosed by a membrane that is an extension of the plasma membrane with which it is contiguous. However, several features inhibit diffusion between the two compartments, such as the high membrane curvature at the ciliary pocket from which the organelle emanates, and the ciliary necklace which appears as a ring of bead-like structures around the base of the cilium. These features allow the ciliary membrane to maintain a lipid and protein composition very different from that of the plasma membrane [46]. Combined with directed trafficking, this allows for the localization and concentration of distinct receptors, ion channels, and other membrane proteins in the ciliary membrane, thereby supporting their sensory and signaling functions. For example, enrichment of the lipid phosphatidylinositol-4-phosphate [PI(4)P] supports the localization of certain GPCRs and ion channels to cilia [49].

## 3. Cilia Serve as Specialized Signaling Organelles

Cilia serve as signaling centers by hosting various proteins and receptors that enable cells to detect and respond to numerous mechanical, chemical, and developmental cues [50,51]. Cilia sense these various inputs and transmit that information to the cell body, thereby regulating diverse cellular responses, preparing downstream signals, and maintaining cellular homeostasis. These organelles also act as a secretory pathway for the release of bioactive molecules, and a growing number of products secreted from cilia are now recognized [3,5,6,52]. A key feature allowing cilia to perform these diverse functions is their ability to concentrate components and receptors in a very small volume, thereby enhancing the signal-to-noise ratio and the capacity to selectively transport proteins and other signaling molecules through the TZ. Key signaling pathways mediated through cilia include Hedgehog, Wnt, mTOR, Hippo, Notch/Delta, platelet-derived growth factor receptor-α (PDGFRα), transforming growth factor-β (TGF-β), autophagy of oral–facial–digital syndrome 1 (OFD1) at centriolar satellites, and the control of primary cilium biogenesis, calcium, and G protein-coupled receptor (GPCR) signaling; this broad array of activities impacts numerous developmental and homeostatic responses and plays essential roles in organismal physiology (reviewed in [16,53]). Motile cilia also can contain both Hedgehog (HH)- and cAMP-dependent signaling proteins [54]. In airway epithelial cilia, hedgehog receptors are not activated by the canonical HH signaling pathway, which involves sonic hedgehog (SHH), that binds to the patched (PTC1) receptor and facilitates GLI transcription factor signaling by removing inhibition imposed on the GPCR smoothened (SMO) by PTC1. Rather, activation occurs through a non-canonical route, where apical SHH decreases intracellular levels of cAMP by involving Gαi and adenylyl cyclase 5/6 and regulates ciliary beat frequency and pH in the airway surface liquid [54]. In addition, the lengths of motile cilia can be controlled by GPCR and TGF-β signaling in various cell types, leading to alterations in their properties [55,56,57].

In the above situations, cilia act to sense some extracellular ligands, the receipt of which is then transmitted to the cell interior. However, there are also situations where they can detect a mechanical signal. For example, cells in the nodes of developing mammalian embryos each bear a single motile cilium that beats in a vortical pattern, setting up a leftward flow in the extra-embryonic fluid. Surrounding these nodal cells is a ring of cells with immotile but mechanosensitive cilia, whose internal structural organization allows for asymmetric bending in response to an applied force. When a unidirectional flow leads to bending of these cilia on one side of the node, the opening of mechanosensitive polycystin channels generates a Ca^2+^ signal that ultimately leads to differential gene expression and the assignment of the left–right body axis [37,38].

## 4. Cilia as Secretory Organelles

**Figure 2:**
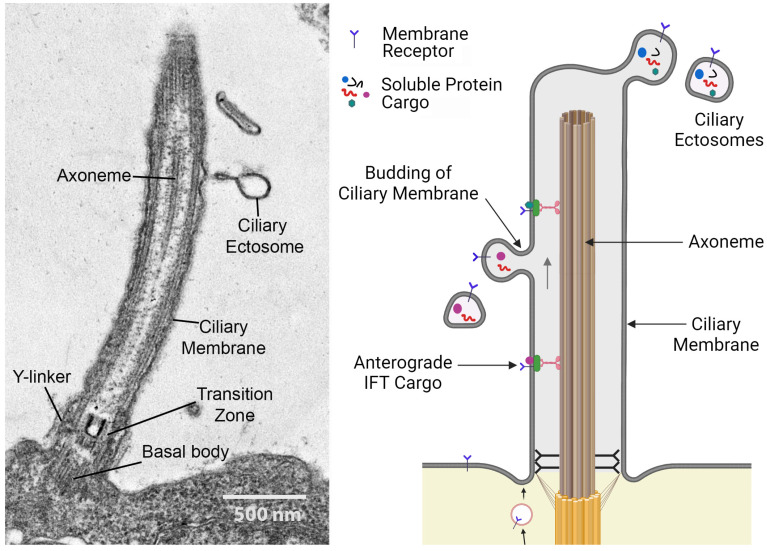
(**Left panel**) Ciliary organization and ectosome release. Longitudinal thin section electron micrograph through a *C. reinhardtii* cilium. The transition zone at the ciliary base acts as a gate to control what components enter the organelle. The ciliary membrane surrounds the microtubular axoneme, which consists of nine outer doublet microtubules and a central pair of singlet microtubules, with their associated structures involved in motility. Nascent ectosome budding from the ciliary membrane is indicated; these contain various factors destined for secretion. Scale bar = 500 nm. (**Right panel**) Diagram illustrating ectosome formation by budding from the ciliary membrane. Membrane proteins and receptors are transported to cilia via Golgi-derived vesicles or through lateral diffusion from the plasma membrane surrounding the cell body. Some of these membrane proteins can be m7oved by the IFT system. Ectosomes form by outward budding of the ciliary membrane and contain various selected membrane proteins and soluble cargoes derived from the cilioplasm. The illustration was created with BioRender.com.

In addition to detecting incoming mechanical and chemical signals, cilia can serve as a conduit for the release of bioactive products into the environment [3,5,6]. This is achieved by the outward budding of the ciliary membrane, releasing extracellular vesicles termed ciliary ectosomes with diameters ranging from ~100 nm to 1 μm (Figure 2) [5].

These can form at the ciliary tip or along the entire length of the cilium (reviewed in [58]). The membrane topology of ectosome budding is such that proteins on the external face of the ciliary membrane will appear on the outer surface of the ectosomes contacting the environment, while their luminal contents are derived from the cilioplasm (i.e., the non-axonemal components inside the ciliary membrane). The release of ciliary ectosomes is evolutionarily conserved and has been observed in the chlorophyte alga *C. reinhardtii*, the nematode *Caenorhabditis elegans*, and mammals [3,6]. Ciliary ectosomes act as long-distance signaling devices, mediating communication between cells, tissue, and organisms [7,59,60]; as delivery vehicles for degradative enzymes [3,61]; and even operate as disposal systems, removing unwanted ciliary components such as activated receptors [52]. Ciliary ectosomes can contain a complex bioactive cargo consisting of proteins, lipids, RNA, and even DNA dedicated to particular cellular functions [3,62,63,64,65]. The secretion of soluble components within the ectosomal lumen also protects them from extracellular proteases and other degradative processes.

Although contiguous, ciliary and plasma membranes have very different lipid and protein compositions [49]. Furthermore, the makeup of ectosomes is distinct from the cilium from which it derives, as its formation involves a two-step regulated trafficking process—first, the highly selective regulated entry of proteins into the cilium proper; and second, the regulated targeting of discrete proteins into nascent ciliary ectosomes in response to cellular or environmental cues (reviewed in [66]). The unique structure and compartmentalization of cilia allows for the controlled transport of proteins through the TZ gate to the ciliary membrane and for the selective concentration of signaling molecules and transmembrane receptors in the highly confined ciliary membrane and luminal space. Further sorting of cilia-localized proteins into ciliary ectosomes appears to be mediated by the endosomal sorting complex required for transport (ESCRT) machinery and/or the post-translational modification of certain ciliary proteins [61,67,68,69]. It is also clear that ectosomes with very distinct protein contents can derive from the same cilium, suggesting a complex cilia-localized sorting system [5,61]. However, much further work is needed to define the mechanisms by which ciliary proteins are targeted to particular ectosome types.

## 5. Cilia as a Platform for Peptidergic Signaling

Many studies have demonstrated that cilia receive signals from the extracellular environment and transduce them to result in a controlled cellular response. Peptide hormones control a wide variety of cellular and physiological activities and mediate intercellular communication among eukaryotes. In *C. reinhardtii*, ciliary ectosomes secreted by budding of the ciliary membrane carry both bioactive peptide products and enzymes required for peptidergic signaling [3,5].

Studies in *C. reinhardtii* and mammalian cells first revealed the link between PAM and cilia through the localization of this enzyme to both Golgi and cilia, and by the direct demonstration that isolated cilia from *C. reinhardtii* exhibit peptide amidating activity [27]. Furthermore, bioactive PAM protein and its amidated products have been identified in ciliary ectosomes released during sexual reproduction in *C. reinhardtii*, suggesting an ancient functional connection between cilia and amidated peptide release [5]. Indeed, biochemical studies of one identified amidated product (termed GATI-*amide*) in *C. reinhardtii* revealed that the amidated propeptide precursor was processed to the final amidated product on the ciliary membrane likely during trafficking into nascent ectosomes ([28]; this pathway is discussed in detail below.

In mammals, several GPCRs such as neuropeptide Y receptor 2 (NPY2R), somatostatin receptor 3 (SSTR3), serotonin receptor 6 (HTR6), dopamine receptors, melanocortin 4 receptor (MC4R), melanin-concentrating hormone receptor 1 (MCHR1), and the orphan receptors GPR161 and GPR175 that mediate neuropeptide/peptide hormone signaling have also been localized to neuronal primary cilia [12,13,70,71,72,73,74,75,76]. Selective localization of these GPCRs into cell-type-specific cilia provides specialized signaling compartments capable of receiving peptidergic signals or detecting other extracellular stimuli, altering the conformation of ciliary GPCRs, triggering intra-ciliary signaling cascades, and ultimately transmitting the resulting signals to the cell interior, where they are transduced to a biological response. For example, NPY2R, localized to the primary cilia of selected brain neurons, plays an important role in regulating food intake and energy metabolism in mammals [77]. When the NPY ligand binds to the NPY2R, it activates the alpha subunit of the associated G-protein (Gαi) which, in turn, inhibits adenylyl cyclase activity, thereby reducing cAMP levels inside the cilium [13]. Once activated, NPY2R exits the cilium and returns to the cell body, where it mediates a downstream signaling cascade. The ability of NPY2R to localize to neuronal cilia is of key importance in this energy metabolism regulatory pathway; mice expressing NPY2R lacking the ciliary targeting signal are obese [13]. Pharmacological manipulation of SSTR3 revealed that ciliary neuro-peptidergic signaling modulates synaptic strength and controls neuronal excitability in the postnatal mammalian brain. Indeed, disruption of the ciliary SSTR3 pathway may provide the underlying mechanistic basis for a series of behavioral and cognitive disorders [78].

## 6. The Peptide Amidation Pathway

**Figure 3:**
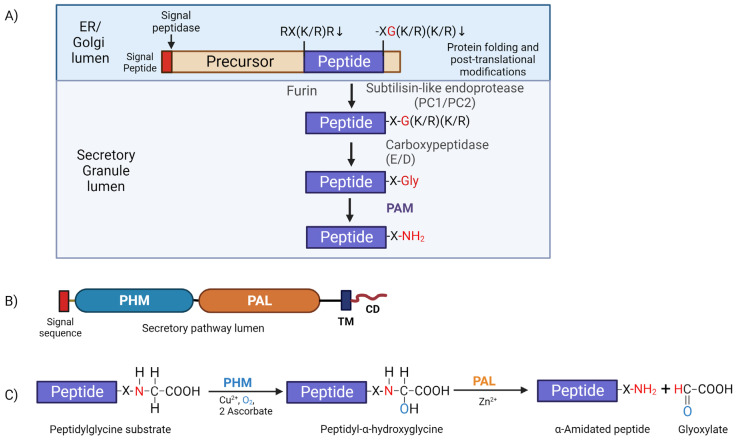
Generalized peptide processing pathway. (**A**) Diagram of a typical peptide precursor (prepropeptide) with an internal furin-like cleavage site (RX(K/R)R) and a canonical amidation site (X-G(K/R)(K/R). In neuroendocrine cells, the signal peptide (red) is removed by signal peptidase in the ER; several post-translational modifications, including N and O-glycosylation, occur as folding proceeds in the ER and mature in the Golgi lumen. When the generally inactive peptide precursor moves from the Golgi into the more acidic lumen of nascent secretory granules, it is further processed. Subtilisin-like endoproteases cleave the precursor at paired basic residues and furin cleavage sites. Carboxypeptidase B-like (CP-E and CP-D) exoproteases then remove exposed C-terminal paired basic residues, yielding a peptidylglycine substrate for PAM. (**B**) Schematic of the PAM protein. The two catalytic cores, peptidylglycine α-hydroxylating monooxygenase (PHM; blue) and peptidyl-α-hydroxyglycine α-amidating lyase (PAL; orange), are connected by a linker region that, in mammals, responds to changes in pH [81]. Both PHM and PAL function in the lumen of the secretory pathway and are anchored by a single-pass transmembrane domain. The small C-terminal cytosolic domain is involved in PAM trafficking through the secretory and endocytic pathways and is also known to bind actin directly. (**C**) The two-step α-amidation reaction catalyzed by PAM. The PHM domain exhibits a strict dependence on copper for its activity and catalyzes the stereospecific α-hydroxylation of the C_α_ carbon of the peptidylglycine substrate. This reaction consumes molecular oxygen and two molecules of ascorbate, which provide the reducing equivalents needed to generate the peptidyl-α-hydroxyglycine intermediate. PAL then cleaves the N-C_α_ bond in a zinc-dependent manner; the N- that was part of the α-NH_2_ group of the peptidylglycine substrate is used to α-amidate the penultimate amino acid and release glyoxylate. The illustration was created with BioRender.com.

The generation of amidated peptides involves a common biosynthetic pathway shared among eukaryotes. Generally, these amidated peptide products are synthesized from large inactive prepropeptide precursors containing a canonical amidation sequence signature (-XG(K/R)(K/R_n_)), where X is the amino acid that will be *α*-amidated in the final product (Figure 3A). Once exposed at the C-terminus, these motifs are first processed by a carboxypeptidase, removing the basic residues, and generating a peptidyl-glycine substrate which can then be acted upon by the bifunctional enzyme peptidylglycine *α*-amidating monooxygenase (PAM) (Figure 3A). This enzyme is a type 1 membrane protein containing two catalytic cores located in the lumen of the secretory pathway, followed by a single-pass transmembrane segment and a small hydrophilic cytosolic domain (CD) located at the C-terminus which act as a routing determinant, controlling the trafficking of PAM protein (Figure 3B). Indeed, the PAM CD can even be cleaved off PAM by *γ*-secretase and directed to the nucleus [79]. Furthermore, PAM protein has an essential role in secretory granule formation in atrial myocytes and acts as a re-usable luminal cargo receptor for atrial natriuretic peptides [80].

PAM-catalyzed α-amidation is a two-step reaction that occurs in the low-pH environment of the secretory pathway (reviewed in [19]). Initially, peptidylglycine *α*-hydroxylating monooxygenase (PHM) catalyzes the hydroxylation of the C*_α_* atom of the exposed C-terminal glycine residue to generate a peptidyl-*α*-hydroxyglycine reaction intermediate; PHM exhibits an absolute requirement for copper, molecular oxygen, and a reducing agent (usually ascorbate). Subsequently, the second enzymatic domain peptidyl-*α*-hydroxyglycine *α*-amidating lyase (PAL) catalyzes cleavage of the N-C*_α_* bond of the hydroxyglycine intermediate in a zinc-dependent manner to generate the *α*-amidated peptide (X-NH_2_) and release glyoxylate as a byproduct [19] (Figure 3C).

After the co-translational removal of the signal peptide and any essential glycosylation, disulfide bond formation, and protein folding, inactive propeptides exit the ER, undergo additional processing while traversing the Golgi stacks, and enter the lumen of immature secretory granules. Lumenal pH and Ca^2+^ levels govern propeptide cleavage by subtilisin-like endo-proteases, which recognize sites with at least two adjacent basic amino acids—Lys(K) and Arg(R)—i.e., (K/R)-(K/R)_n_. Once exposed, these C-terminal basic residues are removed by carboxypeptidase B-like exoproteases (CP-E or CP-D) to generate C-terminal glycine-extended substrates (-X-Gly) for PAM (Figure 3A). Proteins initially synthesized with a C-terminal -Gly act as direct substrates for PAM and do not require processing by endo- or exoproteases. Many potential prepropeptides terminating with a C-terminal Gly have been identified in organisms as diverse as *C. reinhardtii* and scorpions [20,82].

## 7. Regulated Processing and Secretion of Peptidergic Signals through Cilia

Numerous GPCRs that mediate peptidergic signaling have been localized to neuronal cilia, but there is limited information available concerning how these peptide signals are actually trafficked to cilia, detected by GPCRs, and the signaling information finally transduced to the cell body. Studying ciliary signaling in mammals poses significant challenges due to the diversity of cell types, tissues with unique ciliary characteristics, and technical challenges in the isolation and manipulation of cilia and their secretory products associated with ciliary ectosomes. Model organisms such as *C. reinhardtii* and *Caenorhabditis elegans*, where cilia and/or ciliary ectosomes can be isolated and purified, and ciliary signaling readily observed and manipulated, provide a valuable perspective on the evolutionary development of peptidergic signaling through cilia, and act as paradigms to aid our understanding of these processes throughout the metazoans. The enzymes used for the synthesis and processing of amidated peptides are evolutionarily conserved. In mammals, mature amidated peptides are stored in secretory granules in neuroendocrine cells and released through regulated secretion in response to appropriate stimuli. Luminal pH within the secretory pathway plays an essential role in regulating the cleavage of propeptide precursors and storage of the mature peptides [81]. In contrast, *C. reinhardtii*, a unicellular organism surrounded by a multi-layer cell wall, does not contain secretory granules and uses its two cilia to secrete various enzymes such as the subtilisin-like endo-protease VLE1 (vegetative lytic enzyme 1) and amidated products at specific cell and/or life cycle stages [3,5,61,82].

Under nutrient stress conditions, *C. reinhardtii* cells undergo a developmental transition to form sexually competent gametes of two different mating types [83,84]; this life stage alteration is associated with the release of bioactive PAM protein, several α-amidated products, receptors, and subtilisin-like endo-proteases in ciliary ectosomes [5]. A synthetic version of an amidated peptide (GATI-amide) that can be obtained from one of the identified amidated peptide precursors (Cre03.g204500, known as proGATI) regulates the movement of *C. reinhardtii* gametes as it attracts minus- while repelling plus-mating type gametes [5]. Importantly, the non-amidated control peptide has no chemotactic effects. Biochemical tools enable the processing of the large (nominally 91 kDa) proGATI precursor and the synthesis of the amidated bioactive product to be followed. In mammals, cell-type-specific subtilisin-like prohormone convertases are involved in the processing of propeptide precursors; *C. reinhardtii* utilizes the subtilisin-like protease VLE1 as a ciliary prohormone convertase to cleave proGATI [28]. This suggests that *C. reinhardtii* uses cilia and ciliary ectosomes as a platform for peptide processing and secretion. Indeed, ciliary ectosomes were found to carry multiple peptide products generated from a single propeptide precursor that potentially perform diverse cellular functions [28].

Below, we outline the temporo-spatial processing of the proGATI propeptide precursor and the controlled release of amidated ciliary products via ectosomes that were uncovered in *C. reinhardtii*. Investigating this peptide precursor processing pathway within *C. reinhardtii* cilia provides a paradigm for the mechanisms potentially used in multicellular organisms to process and secrete amidated bioactive products from cilia.

## 8. *Chlamydomonas* Has the Machinery Needed to Generate, Secrete, and Detect Peptidergic Signals

The *C. reinhardtii* genome encodes several hundred proteins with the general characteristics of prepropeptide precursors containing subtilisin-like endoprotease cleavage sites [(K/R)(K/R)], furin-like cleavage sites [Rx(K/R)R],] and/or C-terminal -Gly amidation sites [5,82]. Evidence for ninety-nine of these putative propeptides was found in ciliary ectosomes, with seventy-three containing canonical amidation sites [5]. Several proteases, including a subtilisin-like prohormone convertase and carboxypeptidase B-like enzymes that remove C-terminal basic residues and potentially cleave propeptides to generate PAM substrates, were identified in ciliary ectosomes and the ectosome-depleted soluble secretome [5,82]; bioactive PAM enzyme is present on the ciliary membrane and in ectosomes [5,27]. Four subtilisin-like potential prohormone convertases were found in ciliary ectosomes isolated from the cilia of mating *C. reinhardtii* gametes. VLE1 (Cre01.g049950) is a homologue of human prohormone convertase PC7 (PCSK7) and was the only subtilisin-like endoprotease present in both mating ciliary ectosomes and the soluble secretome [5,82]. The other subtilisin-like endo-proteases identified show homology to human PCSK4 (PC4) (Cre03.g145827 and Cre16.g685250), while Cre17.g735450 is more closely related to human PCSK2 (PC2) and PCSK6 (PACE4) [5].

In addition, the *C. reinhardtii* genome encodes numerous receptors belonging to multiple families, including scavenger receptors, TRP channels, blue light receptors, glutamate, and GPCR-like receptors. Fifteen putative receptors were identified in ciliary ectosomes, including several scavenger receptors and one GPCR-like protein. Although trimeric G*_αβγ_* proteins are not encoded by the *C. reinhardtii* genome, a seven-transmembrane GPCR-like receptor is present in ciliary ectosomes and shows homology to human orphan GPCR receptor GPR107, which reportedly binds to neuronostatin, a short peptide derived from the N-terminus of pro-somatostatin [5,66,85]; GPR107 homologues are also present in plants [86]. In sea urchins, scavenger receptors on sperm flagella are known to bind egg-derived amidated sperm-activating peptide-1, that acts as a species/genus-specific chemoattractant [25].

In total, the substrates and enzymes needed for the synthesis of amidated peptide products are present in the cilia and/or ectosomes of *C. reinhardtii*, where they can potentially generate bioactive signaling molecules.

## 9. Ciliary Localization of the Peptide Amidating Enzyme (PAM)

The *C. reinhardtii* genome encodes a bioactive PAM protein that shares many features with the mammalian PAM enzyme [27]. This enzyme mainly localizes to the Golgi in *C. reinhardtii*, with ~7% of total PAM protein present on the ciliary membrane [27]. As the PAM catalytic domains are located in the lumen of the secretory pathway, when trafficked to cilia, they occur along the entire ciliary length facing towards the external environment [27]. This spatial arrangement of PAM catalytic domains implies that any substrates converted to amidated products by ciliary PAM must be located either on the outside of the ciliary membrane or be present in the extracellular environment. Somewhat surprisingly for a trans-membrane protein, PAM is not released from isolated cilia following detergent treatment, but rather remains tightly bound to the ciliary axoneme via uncharacterized interactions that can be disrupted by treatment with 0.6 M NaCl in vitro; this is similar to polycystin-2 [87], another integral transmembrane protein. The ciliary localization of PAM has been conserved in mammals, where it has been found in the primary cilia of mouse embryonic fibroblasts and retinal pigment epithelial cells, as well as the motile cilia of tracheal and ependymal cells, and both the flagellum and acrosome of sperm [27].

Defects in PAM have been linked to ciliary abnormalities in *C. reinhardtii*, planaria, and vertebrates—both mice and zebrafish [88,89,90]. Artificial microRNA knockdown of PAM in *C. reinhardtii* resulted in the formation of ciliary stubs, which exhibited defective transition zones lacking Y-links and accumulated IFT particles and short fragments of singlet microtubules, suggesting a role in ciliogenesis [88]. In addition, inhibition of monooxygenase activity by 4-phenyl-3-benzenoic acid delayed the rate of reciliation in wildtype *C. reinhardtii*, indicating a potential role for amidating activity per se in cilium biogenesis [88]. In planaria, knockdown of bifunctional PAM using double-stranded mRNA led to a slight reduction in ciliary density on the ventral surface. However, this organism also expresses a separate soluble PHM domain, and when both enzymes are targeted together, an almost complete loss of the ventral motile cilia resulted [88]. Loss of PAM also affects ciliogenesis in zebrafish and mice. The *pam*-null zebrafish embryos exhibit a loss of both cilia and microvilli in the pronephros, and some features of primary ciliary dyskinesia, including cyst-like structures on the pronephros and hydrocephalus; they die with massive cardiac edema after about 10 days [89]. PAM knockout is also lethal in mice, and embryos die at embryonic day E14.5 with short or reduced primary cilia in neuroepithelial cells [15]. In contrast, the conditional knockout of PAM in atrial myocytes is viable although they have fewer secretory granules and reduced levels of atrial and brain natriuretic peptides (ANP and BNP). Interestingly, inactive PAM protein that lacks monooxygenase activity can restore secretory granule formation and levels of the pro-ANP precursor in atrial myocytes [80]. These studies in atrial myocytes suggest that PAM might have a role in the assignment of luminal cargoes to secretory granules, and that PAM loss results in the failure of granule formation. Potentially, a similar situation might take place in *C. reinhardtii*, where the absence of PAM could result in the inability to correctly allocate luminal cargoes to pre-ciliary vesicles, impacting ciliary biogenesis. In combination, these studies indicate that PAM is involved in cilia formation and cilia-mediated signaling pathways. However, whether direct PAM-mediated protein–protein interactions (e.g., with the actin cytoskeleton [88]) or PAM-generated amidated products play key roles in ciliogenesis is yet to be determined.

## 10. Regulated Trafficking of PAM, Amidated Products, and a Prohormone Convertase into Ciliary Ectosomes

Under nutrient limiting conditions, vegetative *C. reinhardtii* cells of minus and plus mating types differentiate into gametes and express mating-type-specific genes that enable gamete recognition, cell fusion, and the formation of quadriciliate zygotes [84]. Both vegetative and gametic cells release bioactive ectosomes from their cilia [5,61]. However, the contents and rates of release of ciliary ectosomes during sexual reproduction differ greatly from those of vegetative cells [5]. PAM protein levels are higher in gametes than vegetative cells, and cilia-derived PAM is present on the outer surface of ciliary ectosomes isolated from mating gametes, but is absent from ectosomes obtained from vegetative cell cilia. These observations suggest a function for PAM in sexual reproduction in *C. reinhardtii*. A role for ciliary ectosomes controlling sexual behavior was also reported in Caenorhabditis elegans, where ectosomes released by hermaphrodites alter male mating responses [6].

**Figure 4:**
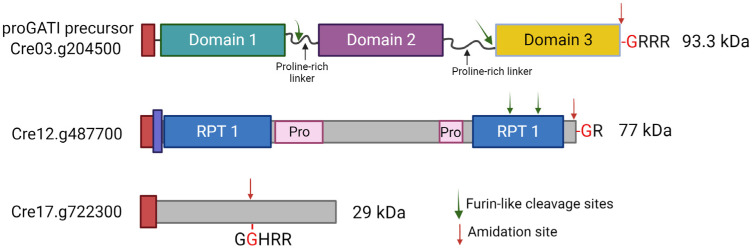
Schematic representation of three prepropeptides identified in *C. reinhardtii*. Domain organizations of the precursors for three amidated peptide products identified by mass spectrometry are shown. All are synthesized with a signal sequence (red) that is presumably removed in the ER. The proGATI protein (Cre03.g204500) is thought to contain three distinct folded domains interconnected by extended proline-rich linkers. Endoproteolytic cleavage at two furin-like cleavage sites produces the N- and C-terminal fragments of proGATI. Amidation at the -GRRR site (red arrow) yields the amidated domain 3 product that acts as a chemotactic modulator [5]. Cre12.g487700, another amidated peptide precursor containing repeat sequences (RPT1, blue) and proline-rich segments (Pro, pink), is also processed to an amidated product of unknown function. This protein contains a predicted transmembrane domain (purple) that partially overlaps the signal peptide sequence (red). The Cre17.g722300 peptide precursor has an amidation site (-GGHRR, red arrow) in the middle of the protein, which requires endoproteolytic cleavage by a subtilisin-like protease followed by exoprotease activity to remove the Arg residues in order to generate a PAM substrate. The illustration was created with BioRender.com.

Subtilisin-like endo-proteases and several amidated peptides were also found in mating ciliary ectosomes. Two amidated peptide precursors (Cre03.g204500 and Cre12.g487700) contain C-terminal amidation sites, requiring only a carboxypeptidase B–like enzyme to generate a PAM substrate. However, a third amidated peptide precursor (Cre17.g722300) would need an endoproteolytic cleavage event, followed by the removal of basic residues to yield a Gly-extended precursor that could be processed by PAM (Figure 4) [5]. The subtilisin-like endo-protease, VLE1, was identified in ectosomes released by the cilia of both vegetative cells, where it acts to degrade the mother cell wall and gametes [5,61]. Direct biochemical analysis using ciliary ectosomes from vegetative cells as a source of VLE1 revealed that *C. reinhardtii* uses subtilisin-like endo-proteases to cleave propeptide precursors, as do metazoans [28].

A 23-mer synthetic peptide (termed GATI-amide) derived from the C-terminus of the proGATI amidated peptide precursor (Cre03.g204500) acts as a chemotactic modulator for *C. reinhardtii* gametes, thereby revealing a chemotactic signaling role for an amidated product secreted through cilia. The control of chemotaxis by amidated peptides has also been reported in Hydra, vespids, and mammals [91,92,93].

## 11. The proGATI Precursor Is Heavily Glycosylated

**Figure 5:**
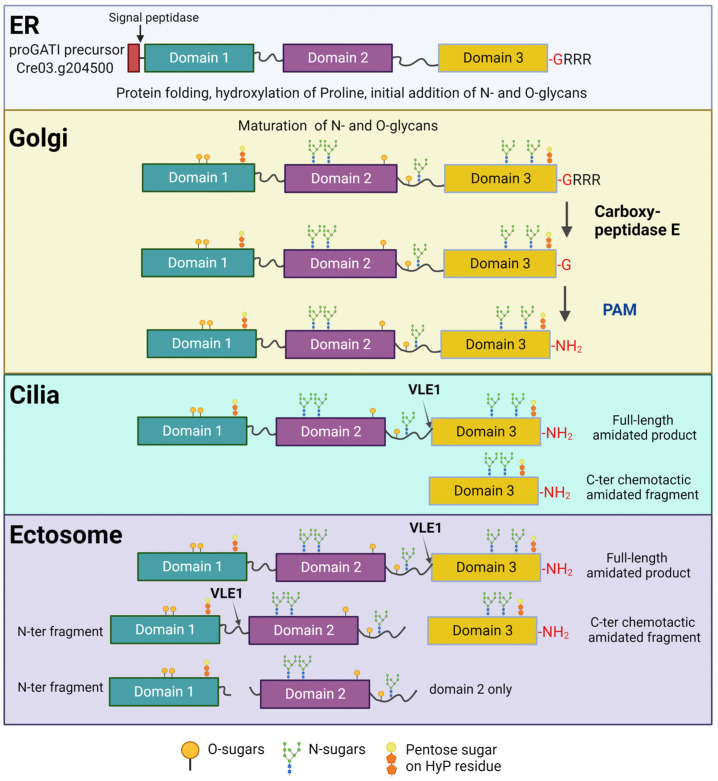
Processing and trafficking pathway of proGATI. Illustration of the processing and trafficking pathway of the proGATI precursor from the ER, through the Golgi and into cilia, where it is ultimately secreted in ciliary ectosomes. In the ER, the signal peptide (red) is removed by signal peptidase and the addition of N- and O-sugars, and hydroxylation of proline (HyP) residues occurs. After transit to the Golgi, the glycans undergo further maturation with the addition of more complex O-sugars at Ser/Thr sites, and pentose sugars are added to the HyP residues. During trafficking through the Golgi, the C-terminal Arg residues (-RRR) are removed by a carboxypeptidase B-like enzyme to generate a C-terminal -Gly (red) extended substrate for PAM that converts it into an α-amidated (-NH_2_) product. This full-length product then enters the cilium, where it is exposed on the external face of the ciliary membrane. Further processing by the VLE1 endoprotease occurs on the ciliary membrane and/or as proGATI is sorted into nascent ectosomes. Biochemical analysis identified the amidated full-length proGATI and cleaved C-terminal chemotactic fragment (domain 3) on cilia, while ectosomes were found to contain N-terminal fragments (either domain 1 alone or joined with domain 2) as well as the full-length protein and C-terminal domain 3 segment; it is unclear whether a single domain 2 fragment is present on ectosomes, as no probe for this domain currently exists. The illustration was created with BioRender.com.

Unlike typical metazoan prepropeptides, pre-proGATI is a large protein with a calculated molecular mass of 93.3 kDa. Structure predictions using AlphaFold (latest v. 2.3.2) and RoseTTAFold (latest v. 3.13) indicate that proGATI contains three distinct folded domains interconnected by long flexible proline-rich linkers (Figure 5). Several N- and O-glycosylation sites were identified in proGATI and due to extensive glycosylation at these locations; the native protein has a much higher apparent molecular mass of ~250 kDa compared with the nominal mass determined from the primary sequence [28]. The N-glycosylation site (N-X-S/T) identified in proGATI is comparable to those found in metazoans, and added glycans can be removed using standard PNGase F treatment that cleaves between the Asn residue and glycan. However, O-glycosylation in plants and algae is distinct from mammals. In these organisms, in addition to the modification of Ser/Thr residues, hydroxy-proline is a major O-glycosylation site and involves the addition of pentose sugars (arabinogalactans) to the hydroxy-proline residues, in a reaction mediated by distinct O-glycosyl transferases [94,95]. Thus, the O-glycans present in proGATI cannot be removed using standard commercially available deglycosylase mixes containing O-glycosidase and neuraminidase. These features underline the differences between proGATI and typical vertebrate peptide precursors.

During synthesis, pre-proGATI is directed into the secretory pathway by a signal peptide that is removed in the endoplasmic reticulum (ER) by signal peptidase. N- and O-glycans are then added to the resulting proGATI as it traffics through the ER and Golgi before transit onto the ciliary membrane. The modified proGATI contains two furin-like cleavage sites; (K^407^PRK) between domains 1 and 2 and (R^693^FSR) at the beginning of domain 3 (Figure 5). Endo-proteolytic cleavage at these sites facilitates the formation of various proGATI fragments on the surface of ciliary ectosomes. These proGATI segments can undergo slow release from the surface of released ectosome, perhaps enhancing their signaling capacity and/or extending their environmental reach, while the full-length proGATI remains tightly bound.

The amidated domain 3 derived from the proGATI precursor contains the C-terminal GATI-amide chemotactic amidated peptide and is released into ciliary ectosomes. The addition of sugars at two potential N-glycosylation sites and a hydroxy-proline O-glycosylation site is probably responsible for the higher-than-predicted molecular mass of domain 3. The glycosylation of peptide hormones is a common phenomenon; for example, the O-glycosylation of several mammalian peptides (such as neuropeptide Y and glucagon family member peptides) is predicted to play a role in propeptide precursor processing, receptor binding, and increased peptide half-life following secretion [96]. Domain 3 of proGATI is resistant to trypsin digestion in vitro, and also exhibits protease resistance on the ectosomal surface [28]. This is unlike many other mammalian hormones that are released into serum and are very short-lived. The resistance of amidated peptides/products to endoproteolytic cleavage is important for stability and both long-distance and long-duration signaling, especially in unicellular organisms where they are exposed to numerous degradative processes.

## 12. Endo-Proteolytic Cleavage of proGATI Is Triggered by Its Ectosomal Entry

Similar to other ciliary proteins, proGATI is synthesized in the cell body and, following post-translational modification in the ER and Golgi, which includes N- and O-glycosylation and C-terminal *α*-amidation after exoprotease removal of several Arg residues, it is specifically trafficked to the ciliary membrane (Figure 5). The cleavage of proGATI into smaller bioactive fragments by mating type-specific subtilisin-like endoproteases occurs on cilia, potentially during their directed trafficking into nascent ectosomes. The fully processed and cleaved 75 kDa domain 3 segment is only found on and released from the cilia of plus gametes [28]. Furthermore, the mating of gametes triggers the movement of this 75 kDa amidated C-terminal fragment into ciliary ectosomes. Analysis of the association of proGATI cleavage products with the ectosomal membrane revealed that each proGATI domain individually associates with the membrane surface and can be slowly released into solution; in contrast, full-length proGATI is bound to the ectosome membrane by three different domains and remains tightly bound [28].

Cell-type-specific subtilisin-like proteases mediate the endo-proteolytic cleavage of peptide precursors in metazoans [97]. In *C. reinhardtii*, the proGATI precursor is cleaved by VLE1, a subtilisin-like endo-protease containing a transmembrane domain, present in cilia and ciliary ectosomes. Mass spectrometry revealed that VLE1 is the only subtilisin present in the cilia of plus gametes, while a distinct subtilisin-like protease also containing a transmembrane domain was found exclusively in cilia and ectosomes from minus gametes [98]. An in vitro assay with recombinant proGATI protein expressed and purified from HEK293 cells demonstrated that it is a direct substrate for VLE1 [28]. In metazoans, luminal pH controls the activation of prohormone convertases. However, with cleavage happening on the ciliary and/or ectosomal surface, the control of luminal pH cannot be a regulatory mechanism in *C. reinhardtii*.

**Figure 6:**
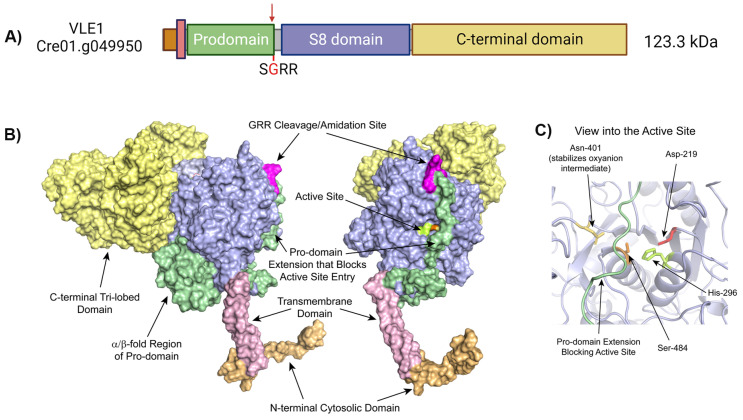
Processing of VLE1, a subtilisin-like protease, in cilia. (**A**) Schematic representation of the VLE1 (Cre01.g049950) protease is shown. The amidation site (-SGRR) at the end of the N-terminal pro-domain is indicated with a red arrow. Mass spectrometry revealed that, following autoproteolytic cleavage, the two Arg residues are removed and the exposed Gly is converted to an α-amide. (**B**) A structural model of VLE1 generated using RoseTTAFold is shown. Two views of the VLE1 molecular surface related by an ~90° rotation about the vertical axis reveal an extension of the N-terminal pro-domain (green) that blocks access to the active site. Autoproteolytic cleavage at the -Gly-Arg-Arg cleavage/amidation site (magenta) located at the junction of the pro-and catalytic domains and subsequent release of the pro-domain activates the S8 enzymatic core of VLE1. (**C**) The active site of VLE1. The pro-domain strand (green) that traverses the active site cleft and blocks substrate entry is shown. The side chains of the catalytic triad residues and the Asn that stabilizes the transition state are also indicated. Panels (**B**,**C**) are reproduced from [28].

The activation of subtilisin prohormone convertases requires the autoproteolytic cleavage of the pro-domain that is needed for folding of the catalytic domain, separating it from the catalytic core (Figure 6). Interestingly, the autoproteolytic cleavage of VLE1 to remove its pro-domain is linked to α-amidation of the newly exposed C-terminus of the pro-domain (Figure 6A,B). On cilia, both the amidated pro-domain and catalytic domains are present; however, activation occurs upon trafficking into ectosomes, where both the pro-domain and transmembrane domain are discarded and only the S8 catalytic domain and C-terminal region are present.

## 13. Conclusions

Cilia provide a platform for the production, transport, release, and reception of peptide signals within a confined space. Secreted peptides are integral to cellular communication and influence a wide variety of physiological processes, including development, neurotransmission, and hormonal regulation. The understanding that cilia play an active role in the production as well as reception of these peptidergic signals redefines paradigms associated with both cilia and peptide biology. Using the model organism *C. reinhardtii* to dissect the complex biochemical pathway by which a peptide precursor is processed, trafficked through cilia, and finally secreted to affect the motility of other cells, using processes clearly related to those employed to produce bioactive peptides that are stored in and released from the secretory granules found in neurons and endocrine cells, provides a basis for understanding and analyzing potential ciliary signaling systems in other organisms. Furthermore, delineating the molecular mechanisms governing the interactions of peptides and cilia may provide insight into their potential roles in the complex phenotypes associated with many ciliopathies.
